# A commentary on ‘Early oral feeding versus nasojejunal early enteral nutrition in patients following pancreaticoduodenectomy: a propensity score-weighted analysis of 428 consecutive patients’

**DOI:** 10.1097/JS9.0000000000001176

**Published:** 2024-02-12

**Authors:** Meijing Li, Mengyi Yin, Xiaobei Jiang, Xiaotong Yang, Lu Liu

**Affiliations:** Hepatobiliary&Pancreatic&Spleen Surgery, Yantai Yuhuangding Hospital, Yantai, Shandong, People’s Republic of China

*Dear Editor*,

It was with great interest that we read the article published in the *International Journal of Surgery*. Jing *et al*.^1^ conducted a retrospective cohort analysis on patients who received early oral feeding (EOF) and nasojejunal early enteral nutrition (NJEEN) following pancreaticoduodenectomy (PD). The study found that EOF is more effective than NJEEN in reducing the incidence of grade B/C DGE (delayed gastric emptying) after PD, implying that the EOF treatment is safe and practicable and should be suggested as the best postoperative feeding strategy after PD.

PD is the only curative treatment for periampullary, pancreatic, biliary tract, and duodenal tumors, and it is linked with a high postoperative morbidity rate of 30–50%, despite significant advances in operating procedures and perioperative care^2,3^. Several studies have identified EOF as the preferred routine feeding approach following PD. EOF can minimize the duration of stay while also significantly increasing nutritional intake in the first 5 postoperative days. There were no significant changes in POPF (postoperative pancreatic fistula), DGE, postoperative morbidity, or death rates between an oral diet, enteral nutrition via NJT (nasojejunal tube), JGT (jejunogastrostomy tube), or DT (duodenal tube), and TPN (total parenteral nutrition) following PD. Enteral nutrition often causes a high rate of dislodged lines and tubes, which might result in times of insufficient nutrition for the patient, as well as additional costs and burden on endoscopic installation. However, EOF reduced postoperative care expenses by reducing the need for the replacement of blocked/infected/dislodged lines and tubes, specialty dietetic teams, and food preparations.

Several considerations should be noted: this was a single-center study with strict inclusion criteria that only included patients from China, which may result in some bias in the results; the surgeon’s experience and postoperative care were not taken into account; and parenteral nutrition (PN) was determined based on the patient’s medical condition, which may also cause bias. It should be highlighted that the use of PN differed significantly among studies. One study offered supplemental PN to all patients, whereas other study commenced PN only when enteral feeding was not tolerated or contraindicated^4,5^.

In conclusion, EOF following PD seems safe based on available data, and it may also have extra benefits related to nutrition, expenses, and patient experience that are currently poorly understood because of a lack of high-quality data. To further explore these possible additional benefits, more prospective research is needed. To further comprehend the nutritional impact of various feeding techniques, future studies in this field should take into account the noteworthy absence of biochemical nutritional indicators, such as albumin, during the postoperative period. Furthermore, there was a dearth of information gauging the post-discharge development of patients, including functional recovery and weight gain.

## Ethical approval

This manuscript is a comment. Don’t need ethical approval.

## Consent

This manuscript is a comment. Don’t need patients consent.

## Author contribution

M.L. and M.Y.: study concept or design, data collection, data analysis or interpretation, and writing the paper; X.J. and X.Y.: data collection; L.L.: study concept or design, writing and revising the paper.

## Conflicts of interest disclosure

This manuscript is a comment without conflicts of interest.

## Research registration unique identifying number (UIN)

This manuscript is a comment. Don’t need UIN.

## Guarantor

Lu Liu.

## Data availability statement

This manuscript is a comment. Don’t need a Data availability statement. However, all the data from the current study are publicly available.

## Provenance and peer review

This manuscript is a comment without being invited.
